# Complex Post-Traumatic Stress Disorder and Borderline Personality Disorder: A Systematic Review of Diagnostic Distinction and Comorbidity

**DOI:** 10.3390/healthcare14162527

**Published:** 2026-08-13

**Authors:** Alejandra Galvez-Merlin, Sandra Diaz-Gonzalez, Esther Julian-Montaner, Noelia Fuentes-Garcia, Myriam Gonzalez-Gomez, Jose Manuel Lopez-Villatoro, Marina Diaz-Marsa, Jose Luis Carrasco

**Affiliations:** 1 Health Research Institute, Hospital Clínico San Carlos (IdISSC), 28040 Madrid, Spain; sanddi04@ucm.es (S.D.-G.); nfuent01@ucm.es (N.F.-G.); jolope09@ucm.es (J.M.L.-V.); marinafd@ucm.es (M.D.-M.); jlcarras@ucm.es (J.L.C.); 2 Department of Legal Medicine, Psychiatry and Pathology, Faculty of Medicine, Universidad Complutense de Madrid (UCM), 28040 Madrid, Spainmyrgonza@ucm.es (M.G.-G.); 3 Biomedical Research Networking Consortium for Mental Health (CIBERSAM), 28029 Madrid, Spain

**Keywords:** Stress Disorders, post-traumatic, borderline personality disorder, diagnosis, differential, Emotional Regulation, shame

## Abstract

**Highlights:**

- CPTSD and BPD are empirically distinguishable but substantially correlated constructs, with stronger discriminant validity under ICD-11 than DSM-5 criteria.
- Affective dysregulation constitutes the primary bridge symptom between CPTSD and BPD symptom networks.
- A stable negative self-concept (CPTSD) versus an unstable self-concept (BPD) is the most consistent differential diagnostic criterion.
- High comorbidity between CPTSD and BPD is associated with greater early interpersonal trauma and functional impairment.
- Shame differentiates the most severe trauma presentations and should be systematically targeted in treatment.

**Abstract:**

Introduction: The recognition of Complex Post-Traumatic Stress Disorder (CPTSD) as a distinct diagnosis in ICD-11 has intensified the need to clarify its boundaries with Borderline Personality Disorder (BPD), given their symptom overlap and shared traumatic origins. Therefore, this systematic review aimed to examine whether CPTSD is distinct from BPD, assess their comorbidity and symptom overlap, identify key criteria for differential diagnosis, and explore therapeutic implications. Method: A systematic search was conducted in PubMed, Scopus, and Web of Science following PRISMA 2020 guidelines. Seven empirical studies (2015–2025) were included, comprising 2574 adults (mean age 40.4 years; 73.3% women). Methods included latent class analysis, structural equation modeling, and network analysis. The methodological quality of the included studies was evaluated independently by two reviewers using the JBI Critical Appraisal Checklist for Analytical Cross-Sectional Studies. Results: Findings consistently supported that CPTSD and BPD are empirically distinguishable, though substantially correlated, particularly within the ICD-11 framework. High symptom co-occurrence was observed, with affective dysregulation identified as the symptom most centrally connecting the two symptom networks. Self-concept emerged as the most robust differentiator: stable and persistently negative in CPTSD versus unstable and fragmented in BPD. Shame was a key affective marker of more severe presentations, and trauma severity, rather than diagnostic category, was associated with symptom variation across studies. Conclusions: CPTSD and BPD are distinct yet frequently co-occurring conditions. Differential diagnosis should focus on self-concept stability, patterns of behavioral dysregulation, and shame. Treatment requires individualized, trauma-informed, and shame-sensitive approaches, with transdiagnostic emotion regulation strategies across presentations. These conclusions should be interpreted with caution, given the predominantly cross-sectional design and methodological heterogeneity of the available evidence.

## 1. Introduction

The publication of the 11th edition of the International Classification of Diseases (ICD-11) in 2018, implemented in January 2022, marked a significant shift in the nosology of trauma-related disorders by recognizing complex post-traumatic stress disorder (CPTSD) as a distinct diagnostic category [1]. This decision reflects decades of clinical and empirical research indicating that the traditional diagnosis of post-traumatic stress disorder (PTSD) does not adequately capture the pervasive consequences of prolonged interpersonal trauma, particularly its effects on emotion regulation, identity, and interpersonal functioning [2]. In contrast, the Diagnostic and Statistical Manual of Mental Disorders [3,4] has not incorporated CPTSD as a separate diagnosis. The coexistence of these two classificatory frameworks has generated conceptual and methodological challenges, limiting comparability across studies and contributing to uncertainty in clinical assessment and diagnosis.

According to ICD-11, PTSD is characterized by three core symptom clusters: (a) intrusive re-experiencing of the traumatic event, (b) persistent avoidance of trauma-related stimuli, and (c) a current sense of threat accompanied by hyperarousal [1]. CPTSD includes these PTSD symptoms but additionally requires the presence of disturbances in self-organization (DSO), encompassing persistent emotion dysregulation, a negative self-concept marked by shame or guilt, and enduring difficulties in establishing or maintaining close relationships. These additional features are considered to reflect the cumulative impact of chronic or repeated interpersonal trauma, often beginning in childhood [1].

The introduction of CPTSD has raised important questions regarding its boundaries with other psychiatric conditions that share impairments in affect regulation and interpersonal functioning, particularly borderline personality disorder (BPD). BPD is defined by a pervasive pattern of instability in self-image, interpersonal relationships, and affect, accompanied by marked impulsivity, with diagnosis requiring at least five of nine DSM-5 criteria, including frantic efforts to avoid abandonment, inappropriate anger, self-injurious behavior, and transient dissociative symptoms [3,4].

Although considerable symptom overlap exists between CPTSD and BPD, similarities alone do not establish that they represent the same clinical construct. Likewise, while exposure to childhood or interpersonal trauma is highly prevalent among individuals with BPD [5,6,7,8], trauma exposure is neither universal nor specific to the disorder. Consequently, the distinction between CPTSD and BPD remains a matter of ongoing debate, with unresolved questions regarding their diagnostic boundaries, the extent of their comorbidity, and whether they represent distinct disorders, overlapping conditions, or different manifestations of trauma-related psychopathology [9].

These unresolved issues have implications beyond diagnostic classification. An imprecise distinction between CPTSD and BPD complicates the interpretation of research findings, hinders the development of valid diagnostic criteria, and may influence clinical decision-making. Although treatment recommendations for the two disorders differ in emphasis (for example, trauma-focused interventions for CPTSD versus treatments prioritizing personality functioning and relational patterns in BPD) such recommendations depend on whether the available evidence supports meaningful diagnostic differentiation.

Against this background, a critical synthesis of the available evidence is warranted. Despite growing interest in CPTSD, its relationship with BPD remains incompletely understood, with findings varying across studies using different diagnostic frameworks and assessment methods. In particular, discrepancies between studies adopting ICD-11 and DSM-5 criteria have limited consensus regarding the discriminant validity of the two constructs and reduced cross-study comparability [1,4].

The present systematic review was therefore conducted to address these unresolved questions by examining: (1) whether CPTSD constitutes a diagnostic entity distinct from BPD; (2) the degree of comorbidity and symptom overlap between the two disorders; (3) the clinical characteristics that most reliably support their differential diagnosis; and (4) the implications of these findings for assessment, treatment planning, and future research.

## 2. Method

### 2.1. Search Strategy

The search strategy was carried out through a systematic review of English-language articles retrieved from the PubMed, Scopus, and Web of Science databases. The procedure followed the 2020 Preferred Reporting Items for Systematic Reviews and Meta-Analyses (PRISMA) guidelines [10].

The descriptors used were “Borderline Personality Disorder,” “Complex Post-traumatic Stress Disorder,” and “Complex Trauma.” Search filtering was performed by searching for keywords in “Abstract” for Scopus and Web of Science, and “Title/Abstract” for PubMed. The search was conducted in February 2025, and English language filters and articles published in the last 10 years were selected in all cases.

For Scopus and Web of Science, these terms were combined with Boolean operators in the following order: (“Borderline personality disorder”) AND (“Complex Posttraumatic Stress Disorder” OR “Complex trauma”).

For PubMed, the search string was expanded to include the corresponding Index Medicus Medical Subject Headings (MeSH) to complement the abstract-based search. The MeSH terms added were “Stress Disorders, Post-Traumatic” and “Borderline Personality Disorder.” The final PubMed query was:

((“Borderline personality disorder”[Title/Abstract] OR “Borderline personality disorder”[MeSH Terms]) AND (“Complex Posttraumatic Stress Disorder”[Title/Abstract] OR “Complex trauma”[Title/Abstract] OR “Stress Disorders, Post-Traumatic”[MeSH Terms])) AND ((y_10[Filter]) AND (english[Filter])).

This combined approach ensured a comprehensive retrieval of relevant literature while maintaining methodological rigor.

### 2.2. Inclusion and Exclusion Criteria

Inclusion criteria for article selection were: (1) original empirical studies published in English; (2) studies published during the last ten years (2015–2025); (3) studies including participants diagnosed BPD according to DSM-IV, DSM-5, ICD-10, or ICD-11 criteria; (4) studies including an assessment of CPTSD using a standardized and validated questionnaire or interview; (5) studies involving exclusively adult samples.

Exclusion criteria were: (1) studies published in languages other than English, (2) studies involving child or adolescent populations, (3) studies including participants without a formal diagnosis of BPD, (4) studies not using standardized and validated instruments for the assessment of CPTSD, (5) doctoral dissertations, (6) systematic reviews and meta-analyses, (7) book chapters or monographs, (8) single-case analyses or case reports.

### 2.3. Study-Selection Process

**Figure 1:**
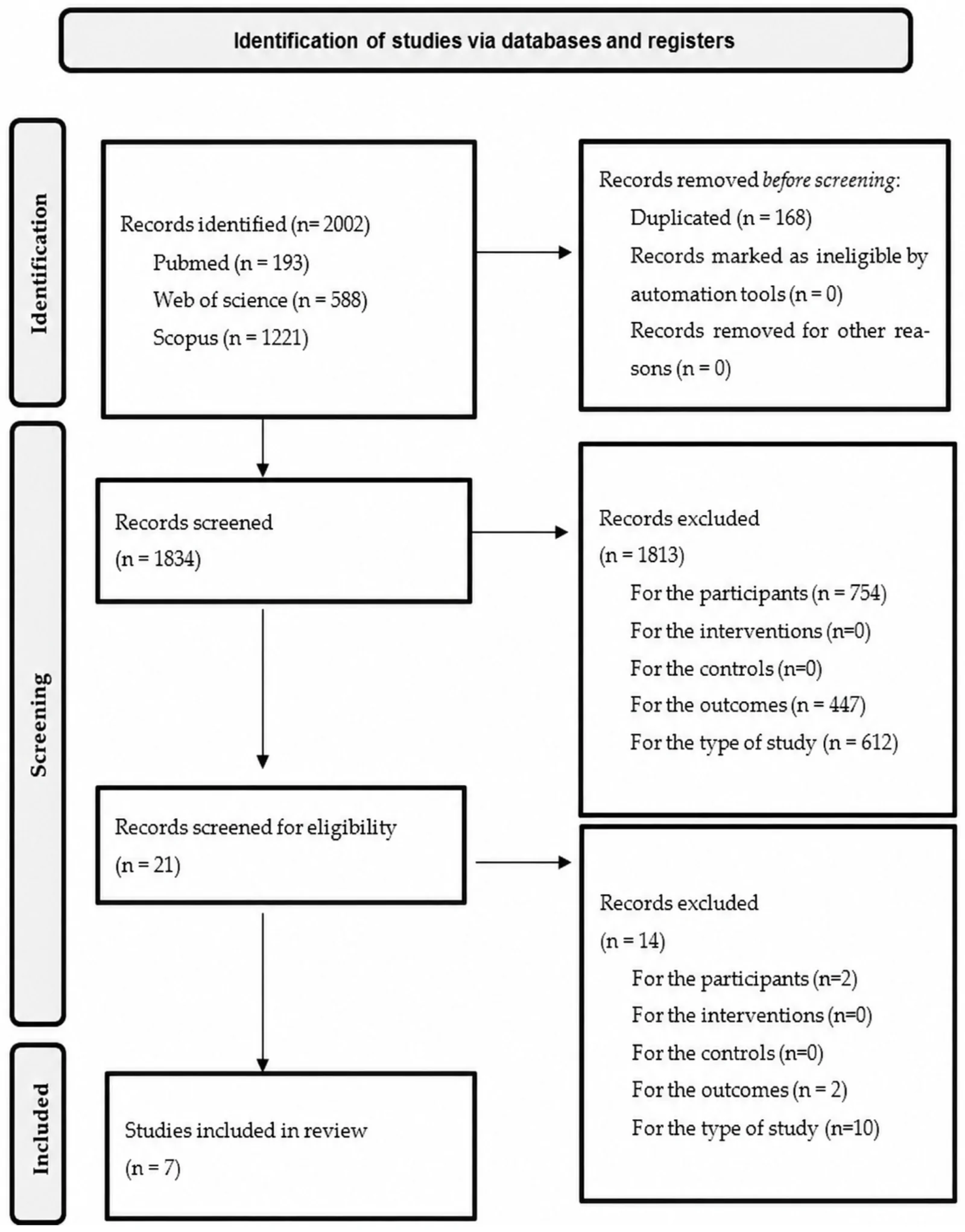
PRISMA flowchart: study selection process.

The study-selection process was conducted in several stages (Figure 1). After the initial identification of records in the databases using the predefined keywords, the Rayyan web (https://www.rayyan.ai/ (accessed on 1 February 2025)) was used for iterative screening and selection: (1) duplicate records were removed; (2) abstracts were screened to retain articles that addressed the target topic—i.e., contained the selected keywords and met all inclusion criteria—while excluding those that failed to meet these requirements or met any exclusion criterion; and (3) full texts of the remaining articles were reviewed to obtain the final set of studies. Title/abstract and full-text screening were both conducted by a single reviewer.

### 2.4. Data Extraction

The characteristics of the studies (short reference of the article, country in which the study was conducted, sample size, % of women, methodology used in the study, instruments used) and the main findings related to the objective of this review were extracted by one reviewer. A second reviewer independently verified the accuracy of the extracted information against the original source articles, resolving any discrepancies by consulting the original article.

### 2.5. Quality Assessment

The methodological quality of the included studies was evaluated using the JBI Critical Appraisal Checklist for Analytical Cross-Sectional Studies [11]. This checklist is widely used and validated for appraising analytical observational research in health sciences. Each item was rated as Yes, No, Unclear, or Not applicable. To address the heterogeneity of analytic designs represented among the included studies (latent class analysis, exploratory structural equation modeling, confirmatory factor analysis, and network analysis), the JBI checklist was supplemented with four additional criteria: (1) model-fit indices reported and adequate; (2) sample size justified for model complexity; (3) measurement invariance or structural equivalence addressed where relevant; and (4) clarity of the diagnostic/symptom ascertainment method. The quality assessment was conducted independently by two reviewers, with disagreements resolved by discussion; item-level ratings for both reviewers are reported in Supplementary Table S1.

## 3. Results

After the initial download of 2002 papers, 7 studies were finally included in the review, adding a total of 2574 people with a mean age of 40.4 years and 73.3% women.

### 3.1. Characteristics of the Studies

The characteristics of the studies finally included in this review are summarized in Table 1 and described below.

The systematic review included seven studies with a total sample of 2574 participants (mean age 40.4 years; 73.3% women). Methodological approaches included Latent Class Analysis (LCA) [12,13], Exploratory Structural Equation Modeling (ESEM) [14,15], Network Analysis (NA) [16,17], and Confirmatory Factor Analysis (CFA) [18].

Regarding assessment, trauma was primarily evaluated using the International Trauma Questionnaire (ITQ) [19], the Childhood Trauma Questionnaire (CTQ) [20], and the Life Events Checklist (LEC) [21] or its updated version, the Life Events Checklist for DSM-5 (LEC-5) [22]. Other specialized tools utilized included the Clinician-Administered PTSD Scale for DSM-5 (CAPS-5) [22], the Posttraumatic Avoidance Behaviour Questionnaire (PABQ) [23], the MINI Scale of Adverse Childhood Experiences (MINI ACE) [24], and the PTSD Checklist for DSM-5 (PCL-5) [22]. Diagnosis of BPD was consistently performed across all studies using the Structured Clinical Interview for DSM-IV Axis II Personality Disorders (SCID-II).

Additionally, secondary clinical domains were measured through instruments such as the Work and Social Adjustment Scale (WSAS) [25], the World Health Organization Five Well-Being Index (WHO-5) [26], the Test of Self-Conscious Affect (TOSCA-3S) [27], and the Inventory of Interpersonal Problems-Circumplex (IIP-C-IRT) [28].

The overall methodological quality, assessed independently by two reviewers using the JBI Critical Appraisal Checklist for Analytical Cross-Sectional Studies, was rated as adequate across the seven included studies; full item-level ratings for both reviewers are reported in Supplementary Table S1.

### 3.2. Methodological Quality of Studies

Methodological quality, assessed independently by two reviewers using the JBI Critical Appraisal Checklist for Analytical Cross-Sectional Studies, was generally adequate across the seven included studies. All seven studies clearly defined inclusion criteria, described study subjects and setting in sufficient detail, measured trauma exposure with valid and reliable instruments, and used statistical analyses appropriate to their respective analytic designs (Items 1, 2, 3, and 8). Item 4 (objective, standard criteria for measurement of the condition) was rated as unclear in six of the seven studies, reflecting a limitation acknowledged by the original authors themselves in five of these six cases: BPD was consistently operationalized through non-validated binary self-report items derived from the SCID-II rather than through a diagnostic interview; Powers et al. [15] was the only exception, having assessed BPD via clinician-administered structured interview. Confounding factors (Items 5 and 6) were identified and addressed in only three of the seven studies, a pattern consistent with the descriptive, classification-oriented nature of latent-variable designs rather than an exposure–outcome framework in which confounding adjustment is standard practice. Regarding the four supplementary criteria applied to address the heterogeneity of analytic designs, model-fit indices (S1) and clarity of diagnostic ascertainment (S4) were adequately reported in all seven studies, whereas sample size was explicitly justified relative to model complexity (S2) in only one study. Notably, measurement invariance or structural equivalence across subgroups (S3) was not assessed in any of the seven included studies, representing the most consistent methodological gap identified in this body of literature. Full item-level ratings for both reviewers are reported in Supplementary Table S1.

### 3.3. Research Findings

The synthesis of the seven included studies provides a comprehensive empirical basis for understanding the relationship between CPTSD and BPD. The findings are organized into three core dimensions: structural validity, clinical differentiation through self-organization and behavior, and the mechanisms underlying their high comorbidity. A summary of the results of the individual studies can be found in Table 2.

#### 3.3.1. Structural Distinction and the Impact of Diagnostic Frameworks

Convergent evidence across multiple statistical methodologies confirms that CPTSD and BPD are empirically distinguishable, though substantially correlated, constructs. Studies utilizing Exploratory Structural Equation Modeling (ESEM) and Confirmatory Factor Analysis (CFA) have consistently identified distinct factorial structures for PTSD, Disturbances in Self-Organization (DSO), and BPD symptoms [14,15,18].

However, this distinction is highly sensitive to the diagnostic framework applied. Research indicates that a clear three-factor model (PTSD, DSO, and BPD) emerges under ICD-11 criteria [15]. In contrast, under DSM-5 criteria, DSO symptoms tend to collapse into the BPD factor, making the two conditions statistically indistinguishable [15]. Furthermore, Network Analysis supports this independence by showing that symptoms organize into structurally separate networks with very few internal connections between the two disorders [16,17]. It should be noted that the one study conducted in a non-clinical/community sample [14] showed comparatively greater overlap between BPD and DSO factors, suggesting that the degree of distinguishability may vary with clinical severity or sampling context.

#### 3.3.2. Clinical Differential Markers: Self-Concept and Behavioral Patterns

The most robust clinical differentiator identified across the literature is the nature of the self-concept. According to Frost et al. (2020) [18], a stable but persistently negative self-concept—characterized by pervasive feelings of failure or worthlessness—is highly specific to the DSO factor of CPTSD. Conversely, BPD is uniquely defined by an unstable and fragmented identity that fluctuates according to relational contexts.

Behavioral dysregulation also follows distinct patterns in each disorder. BPD is specifically associated with externalizing symptoms, such as self-harm, suicidal behavior, frantic efforts to avoid abandonment, and aggressive outbursts [14,15]. In contrast, CPTSD symptoms are more closely linked to internalizing mechanisms, specifically traumatic avoidance, emotional withdrawal, and avoidant attachment styles [15]. While re-experiencing and dissociation are central to the CPTSD network, they do not occupy a structurally equivalent role in the BPD network [16].

#### 3.3.3. Comorbidity, Bridge Symptoms, and the Role of Trauma Severity

Despite being empirically distinguishable, CPTSD and BPD exhibit high rates of symptom co-occurrence, particularly in clinical populations exposed to severe adversity. Latent Class Analysis (LCA) has identified that the most severe clinical profiles are those where both diagnoses overlap [12,13]. These comorbid classes are associated with earlier, more chronic, and multiple interpersonal traumas, as well as greater functional impairment.

Affective dysregulation (encompassing both emotional hyperactivation and hypoactivation or emotional blunting) emerges as the symptom with the highest centrality linking the two symptom networks [17]. Additionally, shame (rather than guilt) has emerged as a central affective marker that distinguishes the most severe comorbid presentations and predicts overall psychological distress better than categorical diagnosis alone [13].

## 4. Discussion

The present systematic review was designed to critically synthesize the available empirical evidence on the relationship between CPTSD and BPD. The findings from the seven included studies allow us to address the four aims established in the introduction: (1) whether CPTSD constitutes a diagnostic entity distinct from BPD; (2) the degree of comorbidity and symptom overlap; (3) the clinical criteria most useful for differential diagnosis; and (4) the therapeutic implications arising from this distinction.

Regarding the first objective of this review, the convergent evidence from studies employing different statistical methodologies consistently supports that CPTSD and BPD are empirically distinguishable, though substantially correlated. Structural approaches, including the exploratory structural equation models of Hyland et al. [14] and Powers et al. [15] and the confirmatory factor analysis of Frost et al. [18], identified distinct factorial structures for PTSD, DSO, and BPD symptoms. These three factors, while correlated, retained distinguishable factor structures, providing robust evidence for the discriminant validity of CPTSD. Network analyses by Knefel et al. [16] and Owczarek et al. [17] further demonstrated that both symptom systems are organized as structurally separate networks with very few bridging connections.

An important qualification must be noted: the degree of distinguishability between the two disorders depends on the diagnostic framework employed. Powers et al. [15] showed that a clear three-factor solution emerges under ICD-11, whereas under DSM-5 criteria DSO symptoms collapse onto BPD, rendering the constructs empirically indistinguishable. This finding lends direct empirical support to the ICD-11 nosological decision to recognize CPTSD as an independent category and has substantive implications for its non-inclusion in the DSM-5-TR.

For the second objective, latent class analyses consistently identified mixed CPTSD–BPD classes as the most prevalent and most severe. Jowett et al. [12] and Saraiya et al. [13] found that the best-fitting models were characterized by high symptomatic co-occurrence rather than by the presence of one diagnosis in isolation.

Affective dysregulation emerges as the principal transdiagnostic domain of overlap across all methodologies. Owczarek et al. [17] identified it as the primary bridge node between both symptom networks, reflecting its centrality within the network structure common to both symptom profiles. However, as a cross-sectional association, this does not establish a mediating or causal role. Hyland et al. [14] similarly found it to be the only symptom loading across all three factors (PTSD, DSO, and BPD), reinforcing its transdiagnostic character. Notably, Saraiya et al. [13] found that trauma severity, rather than categorical diagnosis per se, was the most powerful differentiator of symptom profiles, raising the question of whether both disorders may, in a substantial proportion of cases, represent different expressions of a shared vulnerability shaped by chronic early adversity.

Regarding the third objective of this review, the most robustly supported differentiating criterion is the nature of self-concept. Frost et al. [18] demonstrated that a stable but persistently negative self-concept was highly specific to the DSO factor of CPTSD, whereas an unstable and labile self-concept was the most distinctive feature of BPD. This distinction carries considerable clinical weight: individuals with CPTSD tend to present a coherent but relentlessly negative self-narrative, while those with BPD display a fragmented self-experience that shifts markedly with relational context.

A second differential criterion involves the direction of behavioral dysregulation. Self-harm, suicidal behavior, and fear of abandonment were consistently identified as BPD-specific features [14,15], while CPTSD was more strongly associated with avoidant and withdrawal-based responses. Re-experiencing and dissociation (central within the CPTSD symptom network [16]) do not occupy a structurally equivalent role in BPD. Finally, shame but not guilt was significantly elevated in the most severe comorbid class [13], pointing to this self-conscious emotion as a clinically meaningful marker of trauma severity and CPTSD predominance.

Finally, regarding the fourth objective of this review about the therapeutic implications, the structural distinctiveness of CPTSD from BPD justifies differentiated treatment approaches. For CPTSD, trauma-focused, phase-based interventions are indicated, though the findings underscore that when DSO features are prominent, sustained work on affect regulation and negative self-concept should precede or accompany trauma processing [17,18]. For BPD, approaches targeting identity instability, fear of abandonment, and behavioral dysregulation (such as Dialectical Behavior Therapy (DBT)) remain the primary modality. Notably, none of the included studies were treatment trials; the treatment implications discussed here are therefore cautious clinical extrapolations rather than conclusions directly supported by the reviewed evidence, and readers should consult the separate treatment-outcome literature for each approach.

The high rates of comorbidity and the identification of affective dysregulation as the principal bridging mechanism suggest that transdiagnostic emotion regulation strategies are particularly warranted in mixed presentations. Rather than premature categorical assignment, a dimensional formulation attending to trauma severity, DSO features, and internalizing versus externalizing patterns may better guide individualized treatment planning. The centrality of shame in severe presentations identifies a gap in current protocols for both disorders that future research should address.

These conclusions should nonetheless be considered in light of the methodological quality of the included evidence. The JBI appraisal identified that BPD diagnostic status was ascertained through non-validated self-report items rather than structured clinical interviews in six of the seven studies, and that measurement invariance across subgroups was not tested in any of the included studies. These limitations temper confidence in the generalizability and structural robustness of the discriminant-validity findings reported above, and the conclusions of this review should therefore be regarded as provisional pending replication in samples assessed with validated diagnostic interviews and invariance testing.

Finally, several limitations of the present review should be acknowledged. These relate both to the review methodology and to the current state of the available evidence. From a methodological perspective, this review was not prospectively registered in a protocol repository (e.g., PROSPERO), and both title/abstract screening and full-text assessment were conducted by a single reviewer, which may have introduced selection bias. In addition, the search was restricted to English-language publications. This criterion may have excluded relevant non-English literature, introducing potential language and publication bias, and findings should be interpreted with this constraint in mind. Although three major bibliographic databases were searched (PubMed, Scopus, and Web of Science), the inclusion of an additional database, such as PsycINFO, may have identified further relevant studies. Likewise, extending the search period beyond 2015–2025 might have yielded additional publications. However, this timeframe was deliberately selected to capture studies conducted after the emergence of the current ICD-11 conceptualization of CPTSD, thereby maximizing the methodological and diagnostic comparability of the included evidence.

Several limitations also stem from the available literature itself. First, only seven studies met the eligibility criteria, limiting the generalizability of the findings and precluding a formal meta-analysis. This scarcity likely reflects the relatively recent recognition of CPTSD as a distinct ICD-11 diagnosis and the consequently limited number of studies employing validated CPTSD measures (most notably the ITQ) alongside standardized assessments of BPD. Second, substantial methodological heterogeneity was observed across the included studies. Analytical approaches included latent class analysis, structural equation modeling, confirmatory factor analysis, exploratory structural equation modeling, and network analysis. Because these methods address different research questions and rely on distinct statistical assumptions, direct comparison of findings across studies should be interpreted with caution. Third, all included studies employed cross-sectional designs, preventing conclusions regarding causal or temporal relationships between trauma exposure, symptom development, and diagnostic classification. Furthermore, the pooled sample was predominantly female (73.3%), which may limit the generalizability of the findings given documented sex differences in trauma exposure and symptom presentation. Notably, none of the included studies reported data on gender-diverse participants (e.g., non-binary or transgender individuals), and most relied on binary sex/gender categorizations. Consequently, the applicability of these findings to gender-diverse populations remains unclear, and future research should prioritize the inclusion of more gender-heterogeneous samples to determine whether the patterns of CPTSD-BPD symptom overlap observed here generalize across gender identities.

Future research should prioritize harmonized assessment strategies, larger and more diverse clinical samples, longitudinal designs capable of examining diagnostic stability and treatment response over time, and multimethod assessment approaches that reduce measurement bias and facilitate comparisons across studies.

## 5. Conclusions

This systematic review provides converging evidence that CPTSD and BPD are empirically distinguishable, though substantially correlated, constructs that nonetheless co-occur at high rates, particularly in individuals with severe early interpersonal trauma. The ICD-11 framework offers superior diagnostic resolution, and these findings may inform, though do not by themselves establish, future consideration of CPTSD as a formally recognized diagnostic entity in the DSM. Clinically, differential diagnosis should attend to self-concept stability, the direction of behavioral dysregulation, and the presence of shame. Treatment planning requires formulation-driven, trauma-informed approaches that are sensitive to the dimensional and transdiagnostic features of each presentation—not merely its diagnostic label.

## Figures and Tables

**Table 1:** Description of the studies included in the review.

Short Reference	Country/City	*N* Total Sample	Mean Age	Gender (% Woman)	Method	Instruments
Jowett, S., Karatzias, T., Shevlin, M., & Albert, I. (2020) [12]	Scotland	195	41	65.1	LCA	CTQ; ITQ; LEC; SCID-II; WSAS.
Saraiya, T.C. et al. (2021) [13]	New York	197	22.9	71.1	LCA	BSI; IIP-C-IRT; LEC-5; MINI ACE; PCL-5; SCID-II; TOSCA-3S.
Hyland, P. et al. (2019) [14]	United Kingdom	546	47.2	69	ESEM	ITQ; LEC; SCID-II.
Powers, A. et al. (2022) [15]	United States	470	41.4	98.1	ESEM	BQ-S; CAPS-5; ECR-R; PABQ; SCID-II;
Knefel, M. et al. (2016) [16]	Austria	219	58	40.2	NA	CTQ; ICD-TQ; LEC-5; SCID-II.
Owczarek, M. et al. (2023) [17]	Scotland	330	38.8	64.26	NA	CTQ; ITQ; LEC; SCID-II
Frost, R. et al. (2020) [18]	Israel	617	22.4	78	CFA	ITQ; LEC-5; WHO-5

**Table 2:** Results and conclusions in the studies.

Short Reference	Results	Conclusions
Jowett, S. et al., (2020) [12]	The LCA identified three distinct classes: a high CPTSD/BPD class characterized by high symptom scores in both conditions; a moderate CPTSD/BPD class characterized by high scores on PTSD and DSO symptoms and moderate BPD; and a low PTSD/BPD class characterized by PTSD symptoms and low confirmation of DSO and BPD symptoms. Both PTSD classes were associated with greater exposure to multiple interpersonal traumas at an earlier stage of life and exhibited greater functional impairment.	The findings support the idea of a diagnosis of CPTSD as a separate entity, although the features of BPD appear to largely overlap with the symptoms of CPTSD in this sample.
Saraiya, T. C., et al. (2021) [13]	The four-class model provided the best fit: 1. high combination of PTSD, CPTSD, and BPD symptoms; 2. moderate combination of PTSD, CPTSD, and BPD symptoms; 3. PTSD symptoms only; 4. healthy class (low symptoms). The class with a high combination of symptoms showed greater exposure to childhood trauma and adverse experiences, greater interpersonal and emotional dysfunction, significantly higher levels of shame but not guilt, and greater overall psychological distress.	CPTSD and BPD were not clearly distinguished as separate classes, suggesting a high degree of symptom overlap in young people who do not seek treatment. The severity of the trauma (rather than the type of diagnosis) may be the key differentiating factor. Shame appears to be a central emotion that distinguishes more severe presentations of trauma. It is recommended that clinical interventions address not only PTSD symptoms but also emotion regulation, interpersonal difficulties, and shame.
Hyland, P. et al. (2019) [14]	The three-factor model showed the best fit: PTSD, BPD, and DSO. All three factors were significantly correlated, although with a moderate positive correlation. PTSD factor was weakly correlated with the other two factors, while BPD and DSO factors were strongly correlated. Although some symptoms were shared among the three factors (emotional dysregulation), other distinctive symptoms were identified (relationship difficulties in the case of CPTSD and self-harm behaviors in the case of BPD). All three factors were associated with childhood interpersonal trauma.	The current findings support the discriminative validity of CPTSD and BPD symptoms. They also demonstrate how these constructs share important similarities in symptom composition and their exogenous correlates.
Powers, A. et al. (2022) [15]	For ICD-11 PTSD, the three-factor ESEM model was the best fit: Factor 1 was related to ICD-11 PTSD (related to avoidance), Factor 2 was associated with DSO symptoms (related to anxious attachment), and Factor 3 was associated with BPD (related to aggressive behavior). For DSM-5 PTSD, the two-factor ESEM model was the best fit (PTSD and DSO/BPD).	There are distinct and overlapping characteristics of PTSD, CPTSD, and BPD. It is evident that the diagnostic structure of CPTSD needs to be considered a distinct construct, as it implies an additive value to PTSD.
Knefel, M. et al. (2016) [16]	The symptoms of PTSD and C-PTSD were strongly interconnected within both disorders and to a lesser extent between them. BPD symptoms were weakly related to each other. Re-experiencing and dissociation were the most central symptoms.	Mental disorders are not discrete entities. The study supports the idea that CPTSD is distinct from BPD, even though they may share traumatic antecedents. It follows that clinically addressing the most central symptoms (dissociation) will not only alleviate those symptoms but will also affect other related ones.
Owczarek, M. et al. (2023) [17]	The symptoms of BPD and CPTSD were located in separate networks, with very few links between them, with the elements of “affective dysregulation” being the only ones that related to both diagnoses. The “unstable identity” item was the most central.	The results support the discriminant validity of the CPTSD and support its distinctiveness from BPD.
Frost, R. et al. (2020) [18]	The underlying structure of CPTSD and BPD symptoms was best explained by a two-factor model that included a “general” factor (vulnerability to all symptoms) and three “specific” factors (PTSD, DSO, and BPD symptoms). CPTSD symptoms were more easily distinguished from the general factor, while BPD symptoms overlapped more with this general factor. The most distinctive symptoms of CPTSD were a stable negative self-concept and an unstable self-concept in BPD. Most risk factors were associated with the general factor.	CPTSD and BPD share a common underlying structure, although they remain two distinct entities. Specifically, they can be most effectively differentiated based on self-concept symptoms.

Note. Borderline Personality Disorder (BPD); Complex Post-Traumatic Stress Disorder (CPTSD); Diagnostic and Statistical Manual of Mental Disorders, 5th Edition (DSM-5); Disturbances in Self-Organization (DSO); Exploratory Structural Equation Modeling (ESEM); Latent Class Analysis (LCA); Post-Traumatic Stress Disorder (PTSD); International Classification of Diseases, 11th Revision (ICD-11).

## Data Availability

No new data were created or analyzed in this study. Data sharing is not applicable to this article.

## Supplementary Materials

The following supporting information can be downloaded at: https://www.mdpi.com/article/10.3390/healthcare14162527/s1, Table S1: Methodological Quality of the Seven Included Studies [12,13,14,15,16,17,18] following JBI Critical Appraisal Checklist for Analytical Cross-Sectional Studies.
