# Supplementary material for: Complex Post-Traumatic Stress Disorder and Borderline Personality Disorder: A Systematic Review of Diagnostic Distinction and Comorbidity
Source: Healthcare (Basel). 2026 Aug 13;14(16):2527. doi: 10.3390/healthcare14162527 (PMC13512573; doi:10.3390/healthcare14162527)
Supplement: Supplementary file 1 [file healthcare-14-02527-s001.zip › healthcare-4416023-supplementary.pdf]

Table S1

Methodological Quality of the Seven Included Studies following JBI Critical Appraisal Checklist for Analytical Cross-Sectional Studies

Criterion	Jowett et al. (2020) [12]	Saraiya et al. (2021) [13]	Hyland et al. (2019) [14]	Powers et al. (2022) [15]	Knefel et al. (2016) [16]	Owczarek et al. (2023) [17]	Frost et al. (2020) [18]
JBI Critical Appraisal Checklist for Analytical Cross-Sectional Studies							
1. Inclusion criteria clearly defined	Y	Y	Y	Y	Y	Y	Y
2. Study subjects and setting described in detail	Y	Y	Y	Y	Y	Y	Y
3. Exposure measured in a valid and reliable way	Y	Y	Y	Y	Y	Y	Y
4. Objective, standard criteria used for measurement of the condition	U	U	U	Y	U	U	U
5. Confounding factors identified	N	N	Y	U	N	N	Y
6. Strategies to deal with confounding factors stated	N	N	Y	U	N	N	Y
7. Outcomes measured in a valid and reliable way	Y	Y	Y	Y	U	Y	Y
8. Appropriate statistical analysis used	Y	Y	Y	Y	Y	Y	Y
Supplementary criteria (analytic design heterogeneity)							
S1. Model-fit indices reported and adequate	Y	Y	Y	Y	Y	Y	Y
S2. Sample size justified for model complexity	Y	U	U	N	N	N	N
S3. Measurement invariance / structural equivalence addressed	N	N	N	N	N	N/A	N
S4. Clarity of the diagnostic/symptom ascertainment method	Y	Y	Y	Y	Y	Y	Y

Note.
Y = Yes; N = No; U = Unclear or partially met; N/A = not applicable. Methodological quality was assessed with the JBI Critical Appraisal Checklist for Analytical Cross-Sectional Studies and four supplementary criteria addressing the heterogeneity of latent-variable analytic designs (latent class analysis, exploratory structural equation modeling, confirmatory factor/bifactor analysis, and network analysis).
